# Host genetic control on rumen microbiota and its impact on dairy traits in sheep

**DOI:** 10.1186/s12711-022-00769-9

**Published:** 2022-11-24

**Authors:** Guillermo Martinez Boggio, Annabelle Meynadier, Albert Johannes Buitenhuis, Christel Marie-Etancelin

**Affiliations:** 1 GenPhySE, INRAE, ENVT, Université de Toulouse, 24 Chemin de Borde Rouge, 31326 Castanet-Tolosan, France; 2 Center for Quantitative Genetics and Genomics, Aarhus University, Blichers Allé 20, 8830 Foulum, Denmark

## Abstract

**Background:**

Milk yield and fine composition in sheep depend on the volatile and long-chain fatty acids, microbial proteins, vitamins produced through feedstuff digestion by the rumen microbiota. In cattle, the host genome has been shown to have a low to moderate genetic control on rumen microbiota abundance but a high control on dairy traits with heritabilities higher than 0.30. There is little information on the genetic correlations and quantitative trait loci (QTL) that simultaneously affect rumen microbiota abundance and dairy traits in ruminants, especially in sheep. Thus, our aim was to quantify the effect of the host genetics on rumen bacterial abundance and the genetic correlations between rumen bacterial abundance and several dairy traits, and to identify QTL that are associated with both rumen bacterial abundance and milk traits.

**Results:**

Our results in Lacaune sheep show that the heritability of rumen bacterial abundance ranges from 0 to 0.29 and that the heritability of 306 operational taxonomic units (OTU) is significantly different from 0. Of these 306 OTU, 96 that belong mainly to the *Prevotellaceae*, *Lachnospiraceae* and *Ruminococcaceae* bacterial families show strong genetic correlations with milk fatty acids and proteins (absolute values ranging from 0.33 to 0.99). Genome-wide association studies revealed a QTL for alpha-lactalbumin concentration in milk on *Ovis aries* chromosome (OAR) 11, and six QTL for rumen bacterial abundances i.e., for two OTU belonging to the genera *Prevotella* (OAR3 and 5), *Rikeneleaceae_RC9_gut_group* (OAR5), *Ruminococcus* (OAR5), an unknown genus of order *Clostridia UCG-014* (OAR10), and *CAG-352* (OAR11). None of these detected regions are simultaneously associated with rumen bacterial abundance and dairy traits, but the bacterial families *Prevotellaceae*, *Lachnospiraceae* and *F082* show colocalized signals on OAR3, 5, 15 and 26.

**Conclusions:**

In Lacaune dairy sheep, rumen microbiota abundance is partially controlled by the host genetics and is poorly genetically linked with milk protein and fatty acid compositions, and three main bacterial families, *Prevotellaceae*, *Lachnospiraceae* and *F082*, show specific associations with OAR3, 5, 15 and 26.

**Supplementary Information:**

The online version contains supplementary material available at 10.1186/s12711-022-00769-9.

## Background

Ruminants can digest plant fiber thanks to the symbiotic microbiota in their rumen. This complex microbial community is composed mainly of bacteria but also includes archaea, fungi and protozoa. Bacteria degrade and ferment fibrous feedstuffs to produce volatile fatty acids (FA), microbial proteins and vitamins and to transform dietary lipids, all of which are used by the animal for maintenance, growth and lactation [1]. The rumen microbiota has been shown to be associated with production traits in dairy cows [2–4] and, more recently, in dairy sheep [5]. Furthermore, some authors have demonstrated that rumen microbiota abundance is under low to moderate control by the host genome [2, 4, 6], which may provide the opportunity to select animals with specific microbial communities that are associated with dairy traits. However, to date, there are no published studies on the genetic association between the rumen microbiota and milk traits in sheep.

In dairy sheep, fine milk composition traits are extremely important for the production of high-quality cheese. The FA present in milk influence its texture and nutritional value, and the proteins influence its coagulation capacity, which in turn affect its heat stability and the cheese yield [7]. Dairy traits in sheep have moderate to high heritabilities (${h}^{2}$) ranging from 0.30 to 0.60 [7, 8], and numerous quantitative trait loci (QTL) and major genes associated with dairy traits have been identified in the ovine genome [7, 9–11]. The detection of QTL contributes to the identification of potential candidate genes and of the underlying mechanisms that determine the genetic expression of these relevant traits. However, for rumen microbiota abundance, there is limited information about possible associated genomic regions. Thus, the identification of QTL that are simultaneously associated with rumen bacterial abundance and milk traits can help reveal the basis of the genetic link and the shared metabolic pathways between the rumen microbiota and dairy traits in sheep.

We hypothesised that, in Lacaune ewes, rumen microbiota abundance is affected by the host genetics and is genetically linked to dairy traits through shared metabolic pathways. Thus, based on the use of the dataset that was previously described by Martinez Boggio et al. [5], the objectives of the current study were to (1) quantify the effect of the host genetics on rumen bacterial abundance by estimating its heritability, (2) identify and quantify the genetic links between rumen bacteria and dairy traits by estimating the genetic correlations between the two, and (3) identify the QTL and potential underlying mechanisms that determine the genetic variation in rumen bacterial abundance and in dairy traits by identifying shared genomic regions between the two through genome-wide association studies (GWAS).

## Methods

### Data structure

Data were obtained from the INRAE Experimental Unit of La Fage (UE 321 agreement A312031, Roquefort, France) between 2015 and 2019. Multiparous Lacaune dairy ewes (mean weight of 77 ± 9 kg) were raised indoors and fed a mixed ration of on average 90% meadow hay and silage plus 10% barley (on a gross matter basis) supplemented with approximately 150 g of a commercial protein concentrate (38% of crude protein on a dry matter basis) distributed in the milking parlor. Adjustment of the percentage of concentrates and forages was done each year according to the feeding value of the forages to cover the needs of the ewes, thus they received the same amount of nutrients during the 5 years. On average during this period, the ewes ingested 3.27 kg of dry matter that contained 16% of crude protein and 30% of crude fiber. The genetic structure of the INRAE La Fage flock consists of two independent groups of ewes, which were both divergently selected, as described by Martinez Boggio et al. [5]. Briefly, genetic selection was based on the estimated breeding values (EBV) for milk somatic cell score (SCS) [8] or on the EBV for milk persistency (PERS), expressed as the coefficient of variation in milk production (CV milk). Ewes belonging to each of these two lines were studied (N = 700). The genetic difference between the ewes from the divergent lines for SCS (SCS+ /SCS−) was 2.19 units of SCS EBV [i.e., a 3.60 genetic standard deviation (SD)], and that for PERS (PERS+/PERS−) was 5.52 units of CV milk EBV (i.e., a 2.10 genetic SD). Ninety-five additional ewes were included in the dataset, which were derived from the oldest SCS line but are currently selected to increase the frequency of the mutant allele (T) of the *suppressor of cytokine signalling 2* (*SOCS2*) gene in the experimental Lacaune population to investigate possible associations with other traits. This allele corresponds to a mutation that was identified in 2015 [12] and explains 12% of the genetic variance in somatic cell count in Lacaune dairy sheep. Thus, the final experimental dataset consisted of data from 795 ewes, including 298 under SCS selection (94 SCS+ and 204 SCS−), 402 under PERS selection (200 PERS+ and 202 PERS−), and 95 under selection for the *SOCS2* mutation.

### Rumen sampling and analysis of the bacterial community

Rumen sampling was performed within 3 days of milk recording. Ruminal contents were sampled from each ewe using a vacuum pump and a medical gastric tube. Then, DNA was extracted and purified from a ruminal sample of 80 μL using the QIAamp DNA Stool Mini Kit (Qiagen Ltd, West Sussex, UK) according to the manufacturer’s instructions, with a previous bead-beating step in a FastPrep instrument (MP Biomedicals, Illkirch, France). The 16S rRNA V3–V4 regions were amplified by a first round of PCR with 30 cycles using the following primers: forward F343 (5′-CTTTCCCTACACGACGCTCTTCCGATCTACGGRAGGCAGCAG-3′; [13]) and reverse R784 (5′-GGAGTTCAGACGTGTGCTCTTCCGATCTTACCAGGGTATCTAATCCT-3′; [14]). Since the Illumina MiSeq technology results in 250-base paired-end reads, we obtained overlapping reads that generated extremely high-quality, full-length reads of the entire V3 and V4 regions in a single run. Single multiplexing was performed using a 6-base pair (bp) index, which was added to the R784 primer, during a second round of PCR with 12 cycles with home-made primers including also Illumina adapters: forward (AATGATACGGCGACCACCGAGATCTACACTCTTTCCCTACACGAC) and reverse (CAAGCAGAAGACGGCATACGAGATGTGACTGGAGTTCAGACGTGT). The resulting PCR products were purified and loaded onto an Illumina MiSeq cartridge (Illumina, San Diego, CA, USA) at the Genomic and Transcriptomic Platform (INRAE, Toulouse, France) according to the manufacturer’s instructions. More details on rumen sampling, DNA extraction and amplicon sequencing are provided in Martinez Boggio et al. [5]. The sequences of the 795 samples were processed using the FROGS 3.0 pipeline [15] as follows: (i) read pre-processing, which consists in the removal of sequences that present a primer mismatch, display an unexpected length i.e., shorter than 300 bp or longer than 500 bp, or that contain at least one ambiguous base; (ii) removal of chimera; (iii) regrouping of sequences by clustering with Swarm in FROGS, and we chose the parameters for a distance equal to 1; (iv) cluster filtering, i.e., removal of the clusters with abundances lower than 0.005% [16]; and (v) taxonomic assignment to operational taxonomic units (OTU) using the SILVA database (version 138) [17] (see Additional file 1: Table S1). The abundance table and the taxonomy files were imported into R (v4.0.2) [18]. The core microbiome was quantified based on the occurrence of OTU across multiple samples, and the proportion of samples over which OTU must occur was set to 90% [19].

### Analysis of dairy traits

Official daily records of milk yield (MY), milk somatic cell count as quantified with a Fossomatic cell counter (Foss, Nanterre, France), and milk fat and protein contents (FC and PC, respectively) were obtained on the 795 adult ewes between 28 and 133 days in milk (DIM). Two milk samples collected per animal during morning and afternoon milking were sent for analysis at the Interprofessional Milk Analysis Laboratory (Agrolab’s Aurillac, France). Milk FC and PC were analyzed with mid-infrared (MIR) techniques on a Milko-Scan™ FT6000 instrument (Foss, Nanterre, France). Thus, we analyzed MY, FC and PC, with FC and PC taken as averages weighted by morning and afternoon milk yield records.

From these official milk records, the MIR spectra for 563 ewes were retrieved to predict the fine profile of the daily milk proteins, i.e., the four caseins: alpha-S1-casein (α_s1_-CN), alpha-S2-casein (α_s2_-CN), beta-casein (β-CN) and kappa-casein (κ-CN), and two whey proteins: alpha-lactalbumin (α-lactalbumin) and beta-lactoglobulin (β-lactoglobulin), and of the FA, i.e., saturated FA (SFA), such as butyric acid (C4:0), caproic acid (C6:0), caprylic acid (C8:0), capric acid (C10:0), lauric acid (C12:0), and palmitic acid (C16:0), and unsaturated FA (UFA), such as oleic acid (*cis-9* C18:1), rumenic acid (*cis-9 trans-11* C18:2) and alpha-linolenic acid (C18:3*n-3*). Milk proteins and FA are expressed in g per 100 mL as averages weighted by morning and afternoon milk yield records. The MIR prediction accuracy, as estimated by the value of the coefficient of determination (R^2^), were retrieved from Ferrand et al. [20] and Ferrand-Calmels et al. [21] for the milk proteins and FA, respectively. The R^2^ for caseins are higher than 0.82, and for β-lactoglobulin and α-lactalbumin are equal to 0.77 and 0.26, respectively. The R^2^ for the SFA and *cis-9* C18:1 are higher than 0.93, and for *cis-9 trans-11* C18:2 and C18:3*n-*3 are equal to 0.91 and 0.74, respectively. Two additional traits, i.e., average lactation somatic cell score (LSCS) and CV milk were included in the analysis to account for the genetic structure of the population that is formed of two divergent lines for SCS and PERS.

### Genotyping

DNA extraction from blood samples and genotyping were performed for the 795 ewes. Of these 795 ewes, 743 were genotyped using a medium-density single nucleotide polymorphism (SNP) chip (Illumina Ovine SNP50 BeadChip: 54,241 SNPs), 314 at the Laboratoire d’Analyses Génétiques pour les Espèces Animales (Jouy-en-Josas, France) and 429 at Aveyron-Labo (Rodez, France). The remaining 52 ewes were genotyped with a low-density SNP chip (Illumina Ovine SNP15: 16,681 SNPs) at Neogen (Lansing, USA), followed by imputation to a medium-density SNP chip within the framework of the Lacaune dairy sheep genomic selection programme [22]. Genotypes were subjected to quality control, based on minimum call rates of 90% for SNPs and 95% for individuals and on the exclusion of SNPs with a minor allele frequency lower than 5%. The final dataset included 773 genotyped individuals and 35,492 autosomal SNPs. Markers were positioned on the 26 *Ovis aries* (OAR) autosomes and mapped to the *Ovis aries* genome assembly Oar_v3.1 [23]. The SNP corresponding to the mutation in the *SOCS2* gene was included in the map of OAR3 (129,722,200 bp).

### Statistical analyses

Microbiota abundance data are compositional data [24]. The abundances of each sample are constrained to a total sum imposed by the sequencing technology used. Therefore, the information is contained in the ratios between OTU abundances, and the raw counts are irrelevant. This produces an interdependence between abundances, and they need to be considered as compositional data [25]. Thus, we applied an approach based on compositional data theory [26] which consisted in transforming the counts into centered log-ratios. As zeros are not compatible with the log-ratios transformation, the OTU abundance data were zero imputed with the geometric Bayesian-multiplicative method [27] through the cmultRepl function of the zCompositions package [28] in R (v4.0.2) [18]. Then, they were centered log-ratio (CLR) transformed with the function clr of the compositions package [29] in R (v4.0.2) [18] and standardised to a variance equal to 1. Hereafter, OTU abundance refers to CLR-transformed abundance data.

### Estimation of variance components

Environmental factors were tested by analyzing the variance of each trait. Statistical significance was defined at P < 0.05. Environmental factors that were significant for the abundance of more than 10% of the OTU were included in the model (Table 1). All traits were tested for the year of sampling (five levels: 2015 to 2019), the number of lactations (three levels: 2, 3, and 4 or more lactations) and litter size (two levels: 1 and 2 or more lambs) as fixed effects, and DIM (28 to 133 DIM) as a covariable. OTU abundances were also tested with the sequencing run for DNA samples (six levels), total sequence number per DNA sample (five levels: ≤ 5000; > 5000 and ≤ 10,000; > 10,000 and ≤ 15,000; > 15,000 and ≤ 20,000; and > 20,000 sequences), time of rumen sampling (morning and afternoon) and the order in which the animal was rumen-sampled (eight levels) as fixed effects. For LSCS and CV milk, which are expressed on a lactation basis, the number of milk recording controls was included as a fixed effect (four levels: 4 to 6, 7, 8, and 9 test-days).

**Table 1:** Environmental effects included in the animal models for operational taxonomic units (OTU) and dairy traits

Trait	DIM	Year	Year lactation	Litter size	Year run	Total sequences	Year time: order	Test-day
OTU^a^	ns	88%	23%	ns	44%	15%	44%	
Milk yield	†	†	ns	ns				
Fat content	†	†	†	ns				
Protein content	†	†	ns	†				
Milk proteins^b^	†	†	ns	†				
Milk FA^b^	†	†	†	†				
LSCS	ns	ns	ns	†				†
CV milk	ns	†	ns	†				†

*DIM* days in milk, *Year lactation* number of lactation nested within year, *Year run* run of sequencing nested within year, *Total sequences* number of total sequences per DNA sample, *Year time order* sampling order nested in rumen sampling time and year, *FA* fatty acids, *LSCS* lactation somatic cell score, *CV milk* coefficient of variation in milk production

^a^Percentage of OTU with a significant effect

^b^Each model for milk protein and fatty acids includes at least one of the environmental effects

†: The effect is included in the model; ns: the effects are non-significant; blank cell: the effect is not tested

In this study, we accounted for the structure of the population under selection through the use of multiple-trait models [30], including the selected traits (LSCS and CV milk) as the first two traits in each model. Two multiple-trait models were used. First, to estimate heritabilities, we used a three-trait model with the abundance of each OTU or a dairy trait as a third trait, and second, to estimate the genetic correlations between OTU abundance and the dairy traits, we used a four-trait model with the abundance of each OTU and a dairy trait as third and fourth traits, respectively. Genetic correlations with absolute values higher than twice the standard error were considered to differ from zero.

The data were analyzed using the following multiple-trait animal model:

1 $$\left[\begin{array}{c} {\mathbf{y}}_{\mathbf{1}} \\ {\mathbf{y}}_{\mathbf{2}} \\ {\mathbf{y}}_{\mathbf{3}} \\ {\mathbf{y}}_{\mathbf{4}} \end{array}\right] =\left[\begin{array}{cccc} {\mathbf{X}}_{\mathbf{1}} & {\mathbf{0}} & {\mathbf{0}} & {\mathbf{0}} \\ {\mathbf{0}} & {\mathbf{X}}_{\mathbf{2}} & {\mathbf{0}} & {\mathbf{0}} \\ {\mathbf{0}} & {\mathbf{0}} & {\mathbf{X}}_{\mathbf{3}} & {\mathbf{0}} \\ {\mathbf{0}} & {\mathbf{0}} & {\mathbf{0}} & {\mathbf{X}}_{\mathbf{4}} \end{array}\right] \, \left[\begin{array}{c} {\mathbf{b}}_{\mathbf{1}} \\ {{\mathbf{b}}_{\mathbf{2}}} \\ {{\mathbf{b}}_{\mathbf{3}}} \\ {{\mathbf{b}}_{\mathbf{4}}} \end{array}\right] + \left[\begin{array}{cccc} {{\mathbf{W}}_{\mathbf{1}}} & {\mathbf{0}} & {\mathbf{0}} & {\mathbf{0}} \\ {\mathbf{0}} & {{\mathbf{W}}_{\mathbf{2}}} & {\mathbf{0}} & {\mathbf{0}} \\ {\mathbf{0}} & {\mathbf{0}} & {{\mathbf{W}}_{\mathbf{3}}} & {\mathbf{0}} \\ {\mathbf{0}} & {\mathbf{0}} &{ \mathbf{0}} & {{\mathbf{W}}_{\mathbf{4}}} \end{array}\right]\,\left[\begin{array}{c} {{\mathbf{a}}_{1}} \\ {{\mathbf{a}}_{\mathbf{2}}} \\ {{\mathbf{a}}_{\mathbf{3}}} \\ {{\mathbf{a}}_{\mathbf{4}}} \end{array}\right] + \left[\begin{array}{c} {{\mathbf{e}}_{\mathbf{1}}} \\ {{\mathbf{e}}_{\mathbf{2}}} \\ {{\mathbf{e}}_{\mathbf{3}}} \\ {{\mathbf{e}}_{\mathbf{4}}} \end{array}\right],$$

where ${\mathbf{y}}_{\mathbf{1}}$, ${\mathbf{y}}_{\mathbf{2}}$, ${\mathbf{y}}_{\mathbf{3}}$, and ${\mathbf{y}}_{\mathbf{4}}$ are the vectors of observations for LSCS, CV milk, OTU abundance or one dairy trait in the three-trait model, and OTU abundance and one dairy trait in the four-trait model; ${\mathbf{b}}_{\mathbf{1}}$, ${\mathbf{b}}_{\mathbf{2}}$, ${\mathbf{b}}_{\mathbf{3}}$, and ${\mathbf{b}}_{\mathbf{4}}$ are the vectors of fixed effects described in Table 1 for each trait; ${\mathbf{a}}_{\mathbf{1}}$, ${\mathbf{a}}_{\mathbf{2}}$, ${\mathbf{a}}_{\mathbf{3}}$, and ${\mathbf{a}}_{\mathbf{4}}$ are the vectors of additive genetic effects; and ${\mathbf{e}}_{\mathbf{1}}$, ${\mathbf{e}}_{\mathbf{2}}$, ${\mathbf{e}}_{\mathbf{3}}$, and ${\mathbf{e}}_{\mathbf{4}}$ are the vectors of residual effects. ${\mathbf{X}}_{\mathbf{1}}$, ${\mathbf{X}}_{\mathbf{2}}$, ${\mathbf{X}}_{\mathbf{3}}$, and ${\mathbf{X}}_{\mathbf{4}}$ are incidence matrices relating fixed effects to vectors ${\mathbf{y}}_{\mathbf{1}}$, ${\mathbf{y}}_{\mathbf{2}}$, ${\mathbf{y}}_{\mathbf{3}}$ and ${\mathbf{y}}_{\mathbf{4}}$, respectively; ${\mathbf{W}}_{\mathbf{1}}$, ${\mathbf{W}}_{\mathbf{2}}$, ${\mathbf{W}}_{\mathbf{3}}$, and ${\mathbf{W}}_{\mathbf{4}}$ are incidence matrices relating additive effects to vectors ${\mathbf{y}}_{\mathbf{1}}$, ${\mathbf{y}}_{\mathbf{2}}$, ${\mathbf{y}}_{\mathbf{3}}$ and ${\mathbf{y}}_{\mathbf{4}}$, respectively. The assumptions of the model are $\mathbf{a}\sim \text{N}(0,\mathbf{A}\otimes \mathbf{P})$ and $\mathbf{e}\sim \text{N}(0,\mathbf{I}\otimes \mathbf{R})$, where $\otimes$ denotes the direct product between two matrices, $\mathbf{A}$ is the pedigree relationship matrix, $\mathbf{I}$ is an identity matrix, and $\mathbf{P}$ and $\mathbf{R}$ are the genetic and residual variance–covariance matrices for the random additive and residual effects, respectively. The pedigree of the Lacaune breed traced back to five generations of ancestors (N = 4296). The analyses were performed using the BLUPF90+ software with the OPTION method VCE [31] and by including 100 initial rounds of EM-REML to obtain initial variance components. We used a convergence criterion of 1e^−10^ which is the value set by default in the BLUPF90 software.

To test the significance of the heritability estimates for the 2059 OTU, an empirical significance threshold for the null hypothesis of no genetic control was estimated. For OTU, the null hypothesis was obtained by randomly shuffling their abundances among the individuals. We selected two OTU (one with many and one with few zeros) and performed the random shuffling 10,000 times. For each permutation and OTU, we estimated the heritability of OTU abundance using a three-trait model, as presented in Eq. (1). To define an error rate of 5%, we arranged the heritability estimates by increasing order, and retained the lower value of the upper 5% yielding a significance threshold obtained for both OTU of 0.10.

In order to determine whether certain bacterial genera were over- or under-represented among the OTU that had a heritability significantly different from zero compared to all the OTU, we tested the percentage of OTU of the same genus in both groups using a Fisher's exact test at P < 0.05.

### Genome-wide association studies

Genome-wide association studies (GWAS) of dairy traits and OTU abundances were performed using the single-step genomic best linear unbiased prediction (ssGBLUP) approach [32]. The following single-trait model was used:

2 $$\mathbf{y}=\mathbf{X}\mathbf{b}+\mathbf{W}\mathbf{g}+\mathbf{e},$$

where $\mathbf{y}$ is the vector of observations for OTU abundance or a dairy trait, $\mathbf{b}$ is the vector of fixed effects described in Table 1, $\mathbf{g}$ is the vector of additive genetic effects, and $\mathbf{e}$ is the vector of residual effects. $\mathbf{X}$ and $\mathbf{W}$ are incidence matrices for $\mathbf{b}$ and $\mathbf{g}$, respectively. The assumptions of the model are $\mathbf{g}\sim \text{N}(0,\mathbf{H}{\upsigma }_{\text{g}}^{2})$, where $\mathbf{H}$ is a matrix that combines pedigree- and genome-based relationships [33] and ${\upsigma }_{\text{g}}^{2}$ is the additive variance, and $\mathbf{e}\sim \text{N}(0,\mathbf{I}{\upsigma }_{\text{e}}^{2})$, where $\mathbf{I}$ is an identity matrix and ${\upsigma }_{\text{e}}^{2}$ is the residual variance. The joint pedigree-genomic relationship matrix $\mathbf{H}$ was constructed as follows:

3 $$\mathbf{H}=\left(\begin{array}{cc}{\mathbf{A}}_{\mathbf{11}}-{\mathbf{A}}_{\mathbf{12}}{\mathbf{A}}_{\mathbf{22}}^{\mathbf{-1}}{\mathbf{A}}_{\mathbf{21}}+{\mathbf{A}}_{\mathbf{12}}{\mathbf{A}}_{\mathbf{22}}^{\mathbf{-1}}\mathbf{G}{\mathbf{A}}_{\mathbf{22}}^{\mathbf{-1}}{\mathbf{A}}_{\mathbf{21}} & {\mathbf{A}}_{\mathbf{12}}{\mathbf{A}}_{\mathbf{22}}^{\mathbf{-1}}\mathbf{G}\\ \mathbf{G}{\mathbf{A}}_{\mathbf{22}}^{\mathbf{-1}}{\mathbf{A}}_{\mathbf{21}} & \mathbf{G}\end{array}\right),$$

which projects genomic relationships $\mathbf{G}=\mathbf{Z}{\mathbf{Z}}^{\mathbf{^{\prime}}}/2\sum {\text{p}}_{\text{i}}(1-{\text{p}}_{\text{i}})$ [34] from genotyped animals (labelled as “2”) to non-genotyped animals (labelled as “1”). The matrix $$\mathbf{A}=\left[\begin{array}{cc}{\mathbf{A}}_{\mathbf{11}} & {\mathbf{A}}_{\mathbf{12}} \\ {\mathbf{A}}_{\mathbf{21}} & {\mathbf{A}}_{\mathbf{22}}\end{array}\right]$$ is the pedigree-based relationship matrix, and the genomic relationship matrix $\mathbf{G}$ is constructed as ${\mathbf{G}}=(1-{\upalpha})\left({\text{a}}+{\text{b}} \frac{{ {\mathbf{ZZ}}^{\prime} }}{{ 2\sum{{\text{p}}_{\text{i}}}{{\text{q}}_{\text{i}}} }}\right)+{\upalpha}{\mathbf{A}}_{\mathbf{22}}$, where $\text{a}$ and $\text{b}$ are chosen to equate average inbreeding and average relationships in $\mathbf{G}$ and ${\mathbf{A}}_{22}$ and ${\upalpha }$ is a small value (0.05) [34–36]. The variance components were estimated using BLUPF90+ with the OPTION method VCE [31].

After solving the ssGBLUP model in Eq. (2), we estimated the SNP effects by back-solving the breeding value estimates as in Eq. (4) [32, 37] and the P value of each SNP as in Eq. (5) [38].

4 $$\left. {\widehat{\text{a}}} \right| {\widehat{\text{g}}} = (1-{\upalpha}){\text{b}} {{\mathbf{Z}}^{\prime}} \frac{1}{2\sum {{\text{p}}_{\text{i}}} {{\text{q}}_{\text{i}}}}{\mathbf{G}}^{-1}{\widehat{\text{g}}}_{2},$$

$${\text{Pvalue}}_{\text{i}}=2\left(1-\Phi \left(\left|\frac{{\widehat{\text{a}}}_{\text{i}}}{\text{sd}({\widehat{\text{a}}}_{\text{i}})}\right|\right)\right),$$

5 $$\text{Var}({\widehat{\text{a}}}_{\text{i}})=\frac{1}{2 \sum {{\text{p}}_{\text{i}}}{{\text{q}}_{\text{i}}}}\left(1-{\upalpha }\right){\text{bz}}_{\text{i}}^{\prime}{{\mathbf{G}}^{-1}}\left(\mathbf{G}{\upsigma}_{\text{g}}^{2}-{\mathbf{C}}^{{\mathbf{u}_{\mathbf{2}}}{{\mathbf{u}}_{\mathbf{2}}}}\right)\times {\mathbf{G}}^{-1}{\text{z}}_{\text{i}}\left(1-{\upalpha}\right)\text{b}\frac{1}{2 \sum {{\text{p}}_{\text{i}}}{{\text{q}}_{\text{i}}}},$$

where $\widehat{\text{a}}$ are the estimates of SNP effects, ${\widehat{\text{a}}}_{\text{i}}$ is the estimate for each SNP $\text{i}$, $\widehat{\text{g}}$ are estimates of breeding values, and the ${\mathbf{C}}^{{\mathbf{u}}_{\mathbf{2}}{\mathbf{u}}_{\mathbf{2}}}$ matrix contains the prediction error covariance of EBV for genotyped animals. The SNP effects and P values were computed using BLUPF90+ and POSTGSF90 [31].

We corrected for multiple testing by using the false discovery rate (FDR) in the p.adjust package in R (v4.0.2) [18]. A genome-wide SNP significance threshold of P < 0.10 and a suggestive threshold of P < 0.30 were applied to each trait. After identification of the lead SNP in a given region, significant SNPs that were located less than 1000 mega base pairs (Mbp) apart and were included in the upper third of the peak were grouped within the same QTL region. The CMplot package in R (v4.0.2) [18] was used to generate Manhattan plots showing the − log_10_(P value) on the y-axis.

We estimated the allele substitution effect for significant SNPs that were detected on the same chromosome and at the same position. The following single-trait model was used:

6 $$\mathbf{y}=\text{SNP}+\mathbf{X}\mathbf{b}+ \mathbf{W}\mathbf{a}+\mathbf{e},$$

where $\mathbf{y}$ is the vector of observations for OTU abundance, $\text{SNP}$ is the significant SNP detected for $\mathbf{y}$ as a fixed effect, coded as 0, 1, 2 when the SNP is homozygous for the first allele, heterozygous, and homozygous for the second allele, respectively, $\mathbf{b}$ is the vector of fixed effects described in Table 1 for OTU abundance, $\mathbf{a}$ is the vector of additive genetic effects, and $\mathbf{e}$ is the vector of residual effects. $\mathbf{X}$ and $\mathbf{W}$ are incidence matrices for $\mathbf{b}$ and $\mathbf{a}$, respectively. The assumptions of the model are $\mathbf{a}\sim \text{N}(0,\mathbf{A})$ and $\mathbf{e}\sim \text{N}(0,\mathbf{I})$, where $\mathbf{A}$ is the pedigree-based relationship matrix, and $\mathbf{I}$ is an identity matrix.

Linkage disequilibrium (LD) along the genome was calculated as the squared correlation of allele counts for two SNPs on each chromosome. The proportion of phenotypic variance explained was calculated for each SNP by defining a window variance of 20 adjacent SNPs and for each QTL region defined in this study. The LD and variance explained were calculated using POSTGSF90 [31].

Genes that were identified for the significant SNPs were retrieved from the Ensembl database using the BioMart web interface based on the *Ovis aries* genome assembly Oar_v3.1 [39]. Then the DAVID functional annotation tool [40] was used to analyse the overrepresented Gene Ontology (GO) biological terms, including biological processes, and Kyoto Encyclopedia of Genes and Genomes (KEGG) pathways [41].

## Results

After bioinformatic analysis, 63% of the 9,552,103 initial DNA sequences obtained from the rumen samples of the 795 ewes were retained. The abundance table included 2059 affiliated OTU, represented by 751 to 168,785 sequences, with a median of 1761 DNA sequences per OTU. The finest taxonomic level was the genus level due to an unknown species frequency of 95%.

Overall, the 2059 OTU from the 795 rumen samples were attributed to 11 phyla, 56 families and 112 genera. Expressed as a percentage of the total sequences for all samples, the most representative phyla were *Bacteroidota* (51%), *Firmicutes* (44%), and *Proteobacteria* (3%). At the next taxonomic level, the most abundant families were *Prevotellaceae* (38%) mainly represented by the *Prevotella* genus, *Lachnospiraceae* (18%) represented by 40 genera, *Ruminococcaceae* (9%) mainly represented by the *Ruminococcus* genus, *Oscillospiraceae* (5%) represented mainly by the NK4A214_group, and three families each represented by one genus: *Christensenellaceae* represented by *Christensenellaceae_R-7_group* (5%), and *Rikenellaceae* represented by *Rikenellaceae_RC9_gut_group* (4%). The average percentage of zeros (OTU not detected in the rumen sample) in the OTU abundance table was 37.5%.

### Description of the dairy traits analyzed

The descriptive statistics for the dairy traits analyzed in our study are in Table 2. LSCS and CV milk were included to account for population genetic structure for the variance estimation of each OTU.

**Table 2:** Descriptive statistics and heritability (${h}^{2}$) of dairy traits in Lacaune ewes

	Mean	SD	CV (%)	${h}^{2}$	SE ${h}^{2}$
Traits expressed on a daily basis^a^					
Milk yield (mL)	1946	589	30.3	0.28	0.06
Fat content (g/100 mL)	7.37	1.14	15.5	0.59	0.06
Protein content (g/100 mL)	5.71	0.52	9.1	0.57	0.06
Alpha-S1-casein	1.38	0.15	10.9	0.54	0.08
Alpha-S2-casein	0.66	0.26	39.4	0.68	0.07
Beta-casein	2.10	0.23	10.9	0.41	0.08
Kappa-casein	0.45	0.04	8.9	0.50	0.08
Alpha-lactalbumin	0.13	0.01	7.7	0.36	0.08
Beta-lactoglobulin	0.46	0.50	108.7	0.44	0.08
Butyric acid (C4:0)	0.25	0.03	12.0	0.50	0.08
Caproic acid (C6:0)	0.21	0.03	14.3	0.53	0.07
Caprylic acid (C8:0)	0.20	0.03	15.0	0.55	0.08
Capric acid (C10:0)	0.73	0.12	16.4	0.58	0.07
Lauric acid (C12:0)	0.49	0.08	16.3	0.60	0.07
Palmitic acid (C16:0)	1.96	0.37	18.9	0.54	0.08
Oleic acid (*cis-9* C18:1)	0.80	0.31	38.7	0.44	0.08
Rumenic acid (*cis-9 trans-11* C18:2)	0.04	0.02	50.0	0.45	0.08
Alpha-linolenic acid (C18:3*n-3*)	0.04	0.01	25.0	0.38	0.08
Traits expressed on a lactation basis					
Lactation somatic cell score (LSCS)	3.29	1.50	45.6	0.33 to 0.41^b^	0.07 to 0.09
CV milk	53.93	12.18	22.6	0.19 to 0.26^b^	0.07 to 0.09

*SD* standard deviation, *CV (%)* coefficient of variation expressed in percentage, *SE* standard error

^a^Milk proteins and fatty acids given in g per 100 mL

^b^Mean heritability of LSCS and CV milk estimated for each three-trait model

### Rumen bacterial heritability

The heritability of rumen bacterial abundance ranged from 0 to 0.29 ± 0.07 with a mean of 0.04 ± 0.03 (see Additional file 2: Table S2). Based on these heritability estimates, we found a group of 306 OTU that had a heritability significantly different from 0 (${h}^{2}$ > 0.10, according to the empirical threshold computed) and a mean of 0.15 ± 0.04. Among these 306 OTU, expressed as a percentage of OTU, the main phyla were *Bacteroidota* (61%), *Firmicutes* (34%) and *Spirochaetota* (2%), and the 10 most represented genera are in Table 3.

**Table 3:** Mean and maximum heritability, and genus representativeness levels over the operational taxonomic units (OTU)

Genus	N	Heritability			G1 (%)	G2 (%)
		Mean	SD	Max		
*Prevotella*	92	0.15	0.04	0.27	30*	22
*Christensenellaceae_R-7_group*	21	0.13	0.03	0.21	7	8
*F082/unknown_genus*	21	0.15	0.05	0.26	7*	4
*Rikenellaceae_RC9_gut_group*	21	0.17	0.05	0.29	7	5
*Prevotellaceae_UCG-001*	15	0.14	0.03	0.20	5*	3
*Ruminococcus*	11	0.15	0.06	0.26	4	6*
*Muribaculaceae/unknown_genus*	8	0.15	0.04	0.21	3	3
*Acetitomaculum*	7	0.15	0.04	0.22	2	2
*Treponema*	7	0.13	0.04	0.19	2	1
*Ruminococcaceae/unknown_genus*	6	0.14	0.02	0.17	2	1

*N* number of OTU grouped in each genus, *SD* standard deviation, *G1(%)* genus representativeness levels over OTU with a significant heritability, *G2(%)* genus representativeness levels over all OTU

*(P < 0.05) Fisher’s exact test

The *Prevotella* genus was significantly overrepresented (92 of the 306 OTU) among the OTU with a significant heritability based on Fisher’s exact test (P > 0.05).

### Genetic correlations between rumen bacteria and dairy traits

We estimated the genetic correlation between each of the 306 OTU and each of the 18 dairy traits included in this study. We obtained 5508 genetic correlations of which 301 (i.e., 5%) were significant (i.e., those that were greater than twice the standard error). This corresponds to 96 OTU abundances that had a significant genetic correlation with one or several dairy traits: 81 OTU were correlated with FC and milk FA (see Additional file 3: Table S3), and 56 OTU were correlated with PC and milk proteins (see Additional file 4: Table S4), and 41 of these 96 OTU were correlated with both groups of traits. For MY, we did not find significant genetic correlations with ruminal bacteria.

#### Genetic correlations with milk fat content and fatty acids

**Fig. 1:**
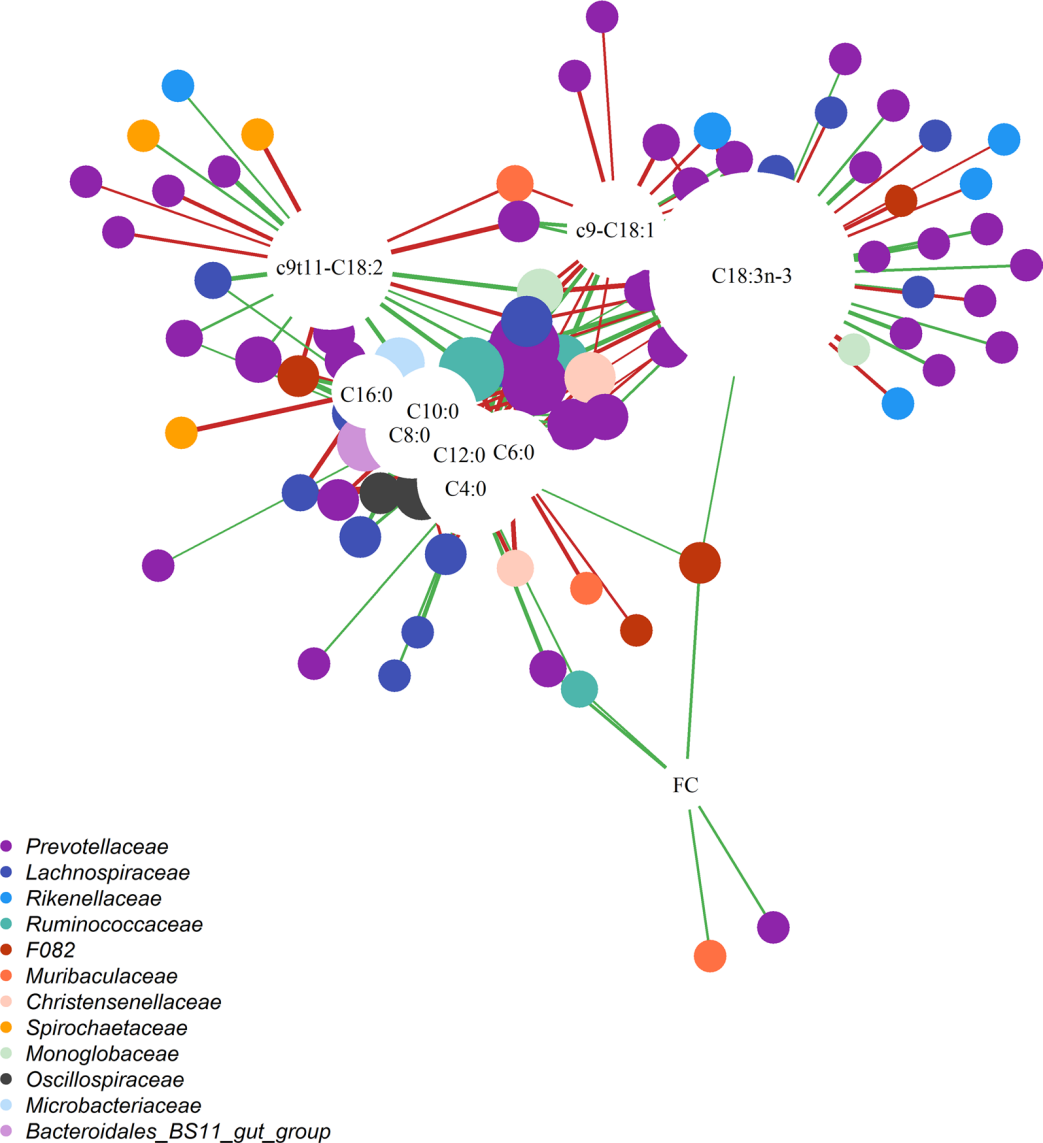
Genetic correlation network between operational taxonomic units (OTU) and fat content (FC) and milk fatty acids, namely, butyric acid (C4:0), caproic acid (C6:0), caprylic acid (C8:0), capric acid (C10:0), lauric acid (C12:0), palmitic acid (C16:0), oleic acid (c9-C18:1), rumenic acid (c9t11-C18:2) and alpha-linolenic acid (C18:3n-3). The nodes represent OTU (in colors associated to the corresponding families where rare families with one OTU are not represented) and dairy traits (in white). The diameter of the nodes is proportional to the number of genetic correlations, and the thickness and color of the edges represent values (− 0.97 to − 0.34 and 0.35 to 0.99) and signs (in green positive and in red negative ones) of the genetic correlation, respectively

Daily fat content and milk FA showed significant genetic correlations with OTU from 26 genera belonging to 17 families. The bacterial families that shared the largest number of significant genetic correlations were *Prevotellaceae*, *Lachnospiraceae* and *Ruminococcaceae* (Fig. 1). The significant genetic correlations ranged from − 0.97 to − 0.34 and from 0.35 to 0.99. Milk SFA shared many more correlated OTU than milk UFA, however C18:3*n-3* was the FA that correlated with the largest number of OTU (42 OTU).

Milk SFA (C4:0 to C16:0) correlated with 41 OTU, of which 26 OTU were positively correlated with at least one SFA. Five OTU were linked with all SFA, with variable genetic correlations, and a closely clustered group consisting of C8:0, C10:0 and C12:0 shared positive correlations with 12 OTU from the *Prevotellaceae*, *Lachnospiraceae* and *Oscilllospiraceae* families (Fig. 1). Milk UFA (*cis-9* C18:1, *cis-9 trans-11* C18:2 and C18:3*n-*3) correlated with 62 OTU, half of which belonged to the *Prevotellaceae* and *Rikenellaceae* families. Two OTU from the *Prevotellaceae* family correlated with all the UFA but showed opposite correlation signs.

#### Genetic correlations with milk protein content and proteins

**Fig. 2:**
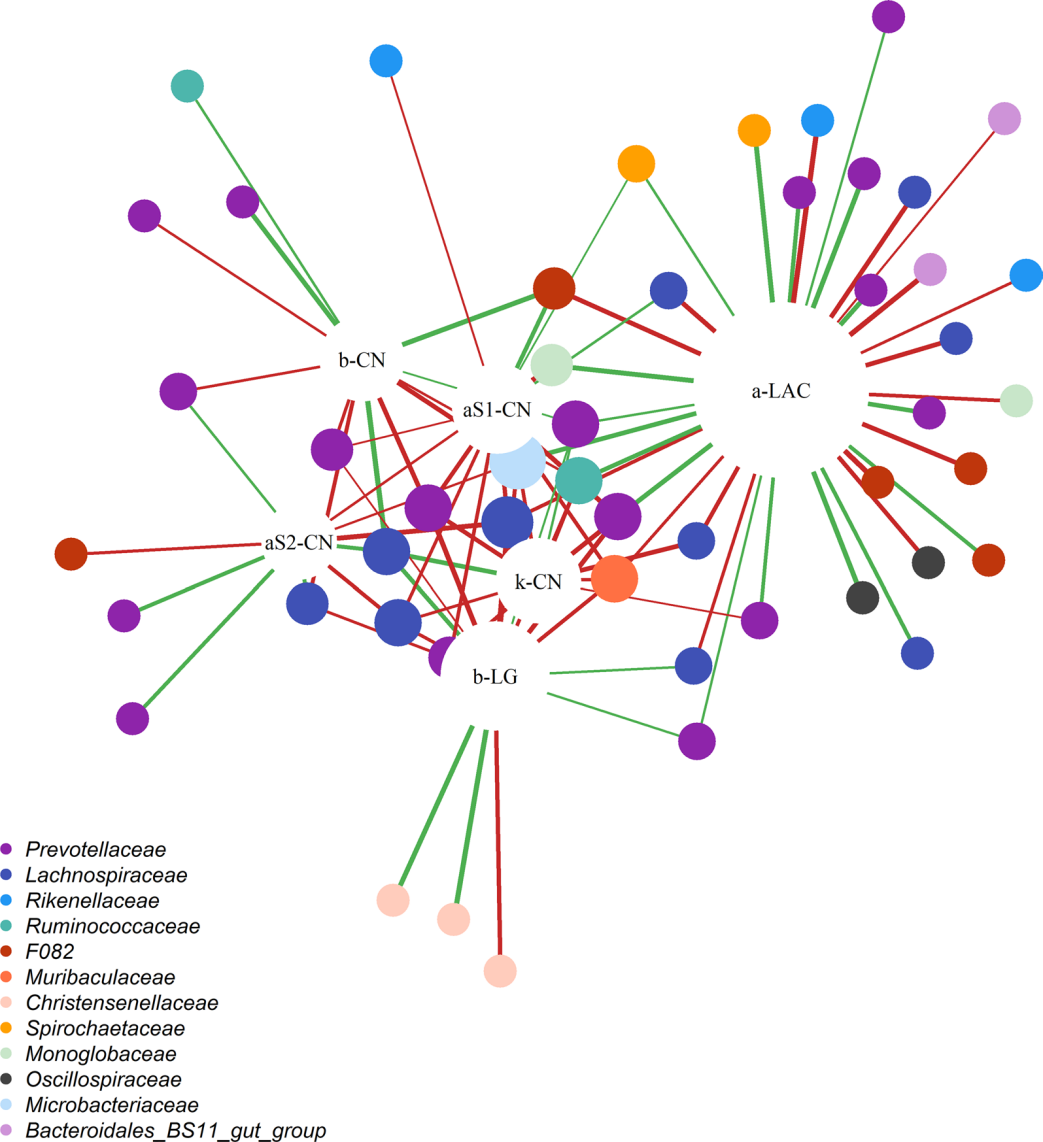
Genetic correlation network between operational taxonomic units (OTU) and milk proteins, namely, alpha-S1-casein (aS1-CN), alpha-S2-casein (aS2-CN), beta-casein (b-CN), kappa-casein (k-CN), alpha-lactalbumin (a-LAC), beta-lactoglobulin (b-LG). The nodes represent OTU (in colors associated to the corresponding families where rare families with one OTU are not represented) and dairy traits (in white). The diameter of the nodes is proportional to the number of genetic correlations, and the thickness and color of the edges represent values (− 0.99 to − 0.33; 0.36 to 0.98) and signs (in green positive and in red negative ones) of the genetic correlation, respectively

The daily protein content and milk proteins showed significant genetic correlations with OTU from 23 genera belonging to 17 families. The bacterial families that shared the largest number of significant genetic correlations were *Prevotellaceae* and *Lachnospiraceae* (Fig. 2). The significant genetic correlations ranged from − 0.99 to − 0.33 and from 0.36 to 0.98. Figure 2 shows two groups of traits: one represented by alpha-lactalbumin, which correlated exclusively with 20 OTU, and a group of caseins (α_s1_-CN, α_s2_-CN, β-CN, and κ-CN) and beta-lactoglobulin, which correlated mostly negatively with OTU.

All caseins were correlated with 30 OTU, of which five were negatively correlated with three or more CN. *Leifsonia* OTU1479 belonging to the *Microbacteriaceae* family showed negative correlations with all CN, and four OTU from the *Lachnospiraceae* and *Prevotellaceae* families were also correlated with two CN (α_s2_-CN and κ-CN). The whey proteins (α-lactalbumin and β-lactoglobulin) correlated with 47 OTU, of which eight were in common. Four OTU from the *Prevotellaceae*, *Lachnospiraceae* and *Muribaculaceae* families showed the same correlation sign with α-lactalbumin and β-lactoglobulin.

### Core microbiome

The core microbiome was represented by 275 OTU, which represent 13% of all OTU, belonging to the most abundant genera, such as *Prevotella*, *Christensenellaceae_R-7_group* and *Ruminococcus*. In the core microbiome, 44 OTU showed a significant heritability (${h}^{2}$ > 0.10), which represented 14% of the heritable OTU, and 13 OTU were genetically correlated with dairy traits.

### GWAS of dairy traits and rumen bacteria

#### GWAS of dairy traits

A GWAS was performed for each of the 18 dairy traits included in this study. The GWAS results are in Table 4, which shows that 22 significant SNPs (FDR < 0.10) were detected for milk yield, alpha-lactalbumin, alpha-S2-casein, caproic acid (C6:0) and caprylic acid (C8:0) concentrations in milk.

**Table 4:** Significant SNPs from the genome-wide association studies of dairy traits

Trait	SNP name	OAR	Position (bp)	Var (%)	− log_10_(P value)
α-Lactalbumin	rs426734075	1	147,595,299	0.06	4.79
α-Lactalbumin	rs400013895	2	25,181,840	0.11	4.41
α-Lactalbumin	rs421261402	2	58,918,403	0.23	4.63
C8:0	rs415371608	2	117,736,662	0.03	5.67
α-Lactalbumin	rs418593908	5	103,652,938	0.04	5.03
α_S2_-CN	rs423428584	6	81,218,896	0.20	8.18
α-Lactalbumin	rs409523937	6	100,115,861	0.22	5.94
α-Lactalbumin	rs399440927	11	32,011,837	0.46	4.43
α-Lactalbumin	**rs429602859**	11	32,652,804	1.05	5.12
α-Lactalbumin	**rs412766461**	11	32,783,984	1.03	4.89
α-Lactalbumin	**rs410865757**	11	33,302,950	1.97	5.40
α-Lactalbumin	**rs402411249**	11	33,379,161	1.75	5.77
α-Lactalbumin	**rs401296484**	11	33,733,902	0.97	5.51
α-Lactalbumin	**rs425950097**	11	34,091,412	0.98	4.43
α-Lactalbumin	**rs420633999**	11	34,146,282	0.88	5.38
α-Lactalbumin	**rs424294429**	11	34,239,169	0.58	4.53
Milk yield	rs402677421	15	7,078,006	0.14	5.82
C8:0	rs405420878	17	51,422,515	0.24	5.79
C6:0	rs405420878	17	51,422,515	0.27	5.60
C6:0	rs410355614	17	52,959,387	0.28	5.30
α-Lactalbumin	rs426375102	20	21,580,756	0.05	4.75
α-Lactalbumin	rs409395180	24	6,819,203	0.07	4.39

SNPs that are located in a quantitative trait locus region are in bold characters

*OAR* *Ovis aries* autosome, *Var (%)* phenotypic variance explained for a window of 20 adjacent SNPs

**Fig. 3:**
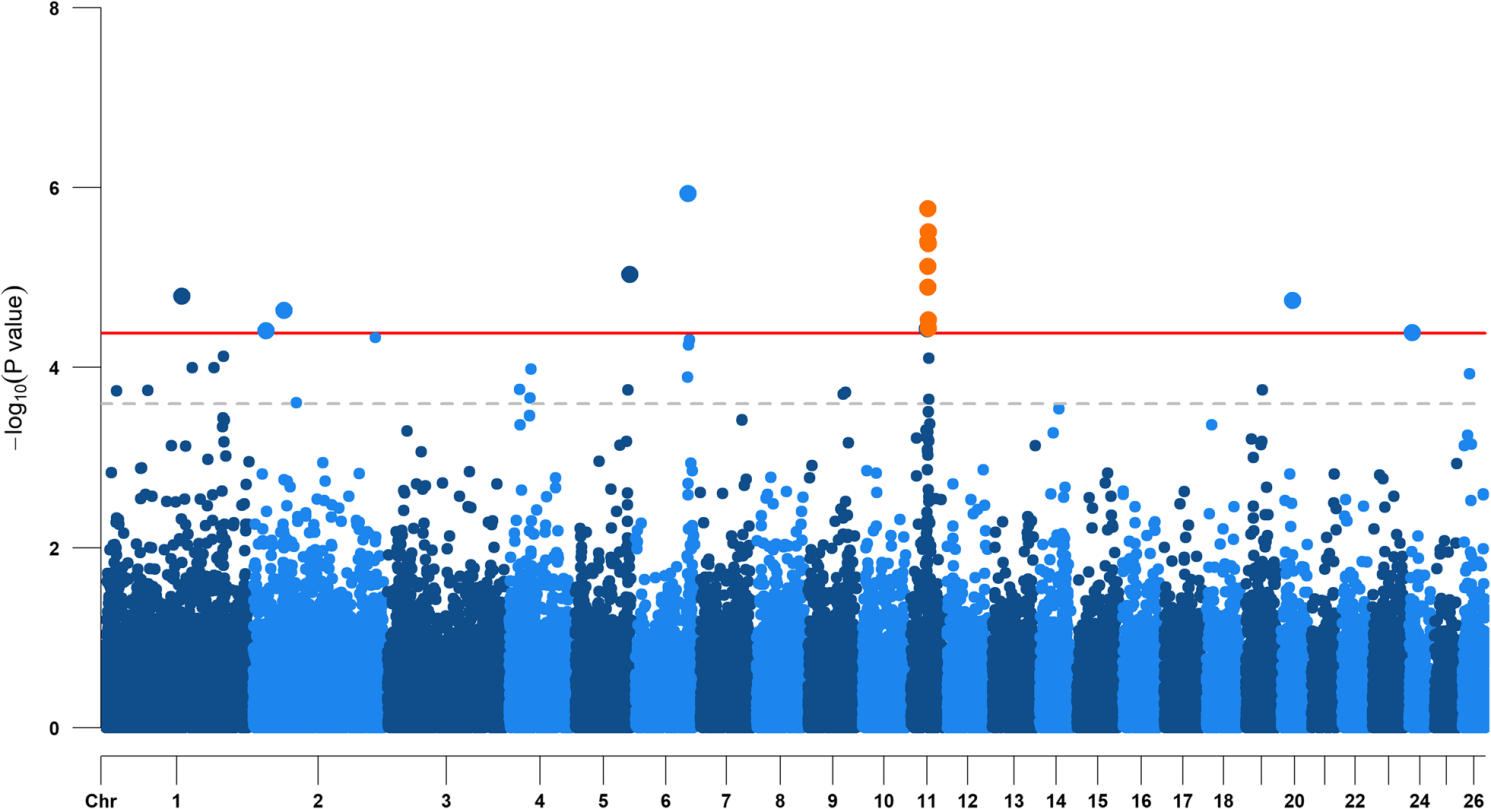
Manhattan plot for the genome-wide association study of alpha-lactalbumin. The horizontal red line and grey dashed line show the false discovery rate thresholds of 0.10 and 0.30, respectively. Orange dots indicate SNPs in the QTL region

The largest number of significant SNPs was detected for alpha-lactalbumin on seven chromosomes, with a QTL on OAR11 (Fig. 3). This QTL on OAR11 includes eight significant SNPs that were located in the genomic region between 32.6 and 34.2 Mbp, with an LD score ranging from 0.24 to 0.52. The QTL region explained 2.5% of the phenotypic variance, with a lead SNP (rs402411249) showing the maximum − log_10_(P value) of 5.77. On OAR6, we detected one significant SNP and one suggestive SNP in the region between 100.1 and 101.3 Mbp with an LD score of 0.56.

For alpha-S2-casein, we detected a significant SNP (rs423428584) on OAR6, with the highest P value (− log_10_(P value) of 8.18) obtained in this study for all dairy traits.

**Fig. 4:**
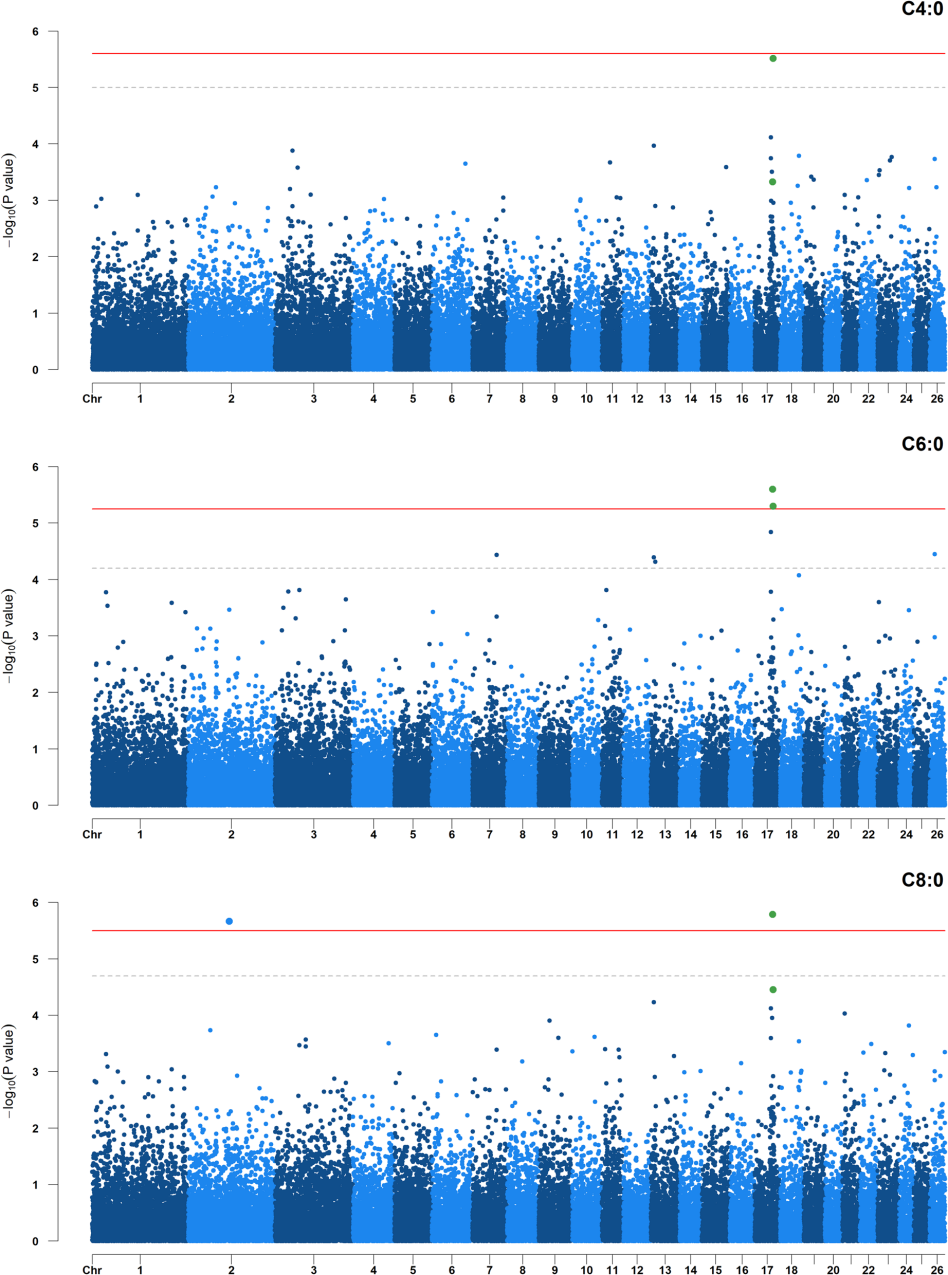
Manhattan plots for the genome-wide association studies of butyric acid (C4:0), caproic acid (C6:0) and caprylic acid (C8:0). The horizontal red line and grey dashed line show the false discovery rate thresholds of 0.10 and 0.30, respectively. Green dots indicate SNPs in common between traits

For the SFA C6:0 and C8:0, we detected the same significant SNP (rs405420878) on OAR17 (Table 4). Another significant SNP (rs410355614) was detected for C6:0 and as a suggestive SNP for C4:0 with a − log_10_(P value) of 5.52 (Fig. 4). However, the LD score between these SNPs was lower than 0.10. For C8:0, a significant SNP (rs415371608) was found on OAR2, which was also identified as a suggestive SNP for C10:0 with a − log_10_(P value) of 5.25.

For milk yield, we detected a significant SNP on OAR15, but we did not identify any signal on OAR3, which included the SNP corresponding to the mutation in the *SOCS2* gene.

#### GWAS of rumen bacteria

A GWAS was performed for each of the 306 OTU abundances that had a heritability significantly different from 0. We detected 94 significant SNPs (FDR < 0.10) that were distributed across 22 chromosomes for 56 OTU (see Additional file 5: Table S5). For the 94 SNPs detected, we recovered 42 potential candidate genes that are involved in various GO biological processes and KEGG pathways (see Additional file 6: Table S6). In addition, as shown in Table 5, six QTL were identified on OAR3, 5, 10 and 11.

**Table 5:** Significant SNPs from the genome-wide association studies of the rumen bacteria on chromosomes that colocalized with dairy traits

Genus	OTU name	SNP name	OAR	Position (bp)	Var (%)	− log_10_ (P value)	Gene name
*Lachnospiraceae_NK4A136_group*	OTU2186	rs422351583	3	5,668,047	0.13	6.09	
*[Eubacterium]_coprostanoligenes_group* /*uG*	OTU2438	rs420593841	3	8,693,890	0.05	5.37	*GARNL3*
*[Eubacterium]_ventriosum_group*	OTU355	rs415665472	3	27,430,018	0.14	5.95	
*Prevotella*	OTU196	rs399829503	3	38,048,434	0.09	5.09	*TIA1*
*Clostridia UCG-014/uF/uG*	OTU2515	rs417512713	3	82,810,037	0.14	5.79	
*Prevotella*	OTU196	rs407226710	3	87,932,154	0.37	5.07	
*Prevotellaceae_UCG-001*	OTU906	rs419358934^a^	3	89,802,120	0.33	6.24	
*Prevotella*	OTU196	**rs419358934**^a^	3	89,802,120	0.18	5.32	
*Prevotella*	OTU196	**rs430243701**	3	89,808,523	0.14	6.78	
*Prevotellaceae_UCG-001*	OTU906	rs424849255	3	99,246,368	0.09	5.25	*IL18R1*
*Prevotellaceae_UCG-001*	OTU906	rs404566475	3	112,090,102	0.23	5.92	
*Syntrophococcus*	OTU509	rs409660427	3	138,657,389	0.14	5.27	*ENDOU*
*Prevotella*	OTU427	rs402872469	3	221,603,160	0.16	5.40	
*Possible_genus_Sk018*	OTU1631	rs402307868^a^	5	31,467,389	0.03	5.76	
*Prevotella*	OTU1843	**rs402307868**^a^	5	31,467,389	0.11	5.11	
*Prevotella*	OTU1843	**rs413547561**	5	31,477,570	0.10	5.99	
*Fibrobacter*	OTU1335	rs405557393	5	59,933,921	0.06	6.10	*ANXA6*
*Rikenellaceae_RC9_gut_group*	OTU546	**rs410315179**	5	72,275,014	0.38	6.75	
*Rikenellaceae_RC9_gut_group*	OTU546	**rs429334236**	5	72,324,510	0.24	5.81	
*Fibrobacter*	OTU1335	rs398177414	5	79,732,944	0.18	6.56	
*Ruminococcus*	OTU1191	**rs411645323**	5	83,683,348	0.20	5.30	
*Ruminococcus*	OTU1191	**rs418870684**	5	83,724,689	0.12	5.29	ENSOARG00000025325
*Lachnospiraceae_UCG-008*	OTU655	rs403619685	6	28,689,782	0.08	5.92	
*Lachnospiraceae_XPB1014_group*	OTU947	rs400970883	6	36,655,091	0.05	6.20	*SPP1*
*Prevotella*	OTU1843	rs416815759	6	45,906,622	1.43	5.59	*TBC1D19*
*Prevotella*	OTU440	rs406261149	6	50,058,344	0.20	5.30	
*Acetitomaculum*	OTU612	rs430647780	6	75,425,021	0.20	5.58	
*Acetitomaculum*	OTU612	rs415319007	6	85,637,777	0.29	5.51	
*Prevotella*	OTU399	rs428296445	10	16,139,981	0.33	5.57	*W5PCY9_SHEEP*
*Clostridia UCG-014/uF/uG*	OTU1386	**rs423990418**	10	16,216,118	0.21	6.28	*W5PCZ6_SHEEP*
*Clostridia UCG-014/uF/uG*	OTU1386	**rs404163943**	10	16,222,665	0.10	6.28	*W5PCZ6_SHEEP*
*Prevotella*	OTU13	rs408448649	10	46,573,078	0.25	5.58	*DACH1*
*Muribaculaceae/uG*	OTU803	rs403822645	10	80,687,261	0.08	4.87	ENSOARG00000026327
*Ruminococcaceae/uG*	OTU155	rs408532009	10	84,678,999	0.35	6.41	*ANKRD10*
*Ruminococcaceae/uG*	OTU501	rs422890024	11	7,826,874	0.04	5.40	*MSI2*
*Christensenellaceae_R-7_group*	OTU596	rs414408260	11	58,151,039	0.14	5.63	
*CAG-352*	OTU304	**rs421085019**	11	61,946,606	0.23	5.48	
*CAG-352*	OTU304	**rs429659626**	11	62,034,261	0.04	5.48	*HELZ*
*Prevotella*	OTU943	rs412819070	15	5,606,060	0.13	5.71	*MMP20*
*Prevotella*	OTU423	rs409362751^a^	15	37,553,506	0.18	6.97	*PDE3B*
*Prevotella*	OTU80	rs409362751^a^	15	37,553,506	0.16	5.84	*PDE3B*
*Prevotella*	OTU399	rs409362751^a^	15	37,553,506	0.19	5.52	*PDE3B*
*Prevotella*	OTU399	rs426932216	15	44,445,054	0.14	5.13	*LOC101105776*
*Blautia*	OTU1025	rs425070593	15	80,043,367	0.20	5.34	*TCN1*
*F082/uG*	OTU167	rs412496804^a^	26	28,316,180	0.31	6.06	
*Prevotella*	OTU1336	rs412496804^a^	26	28,316,180	0.20	5.98	
*Prevotellaceae_UCG-001*	OTU906	rs412496804^a^	26	28,316,180	0.20	5.20	

SNPs that are in a quantitative trait locus region are in bold characters

*OAR* *Ovis aries* chromosome, *Var (%)* phenotypic variance explained for a window of 20 adjacent SNPs, *uF* unknown family, *uG* unknown genus

^a^SNP shared between different OTU

The group of 56 OTU with significant SNPs belonged to three phyla, 11 families, and 23 genera. Expressed as a percentage of OTU, the *Prevotellaceae* family was the most represented, with 23 OTU (41%) of which 19 were *Prevotella* OTU, followed by *Lachnospiraceae* (20%) and *Ruminococcaceae* (9%).

For the 94 significant SNPs detected in the host genome and associated with rumen bacterial abundance, several genomic regions showed QTL and significant SNPs for OTU abundance and dairy traits (Table 5).

#### OAR3

On OAR3, we detected a QTL for *Prevotella* OTU196 that includes two significant SNPs in high LD (LD score of 0.67). The SNP rs419358934 presented a colocalized signal with *Prevotellaceae_UCG-001* OTU906, and the allele substitution effect for that SNP varied in the same direction for both *Prevotellaceae* OTU. In addition, for each OTU, we detected two other significant SNPs that were located outside of the QTL for which two candidate genes were identified (Table 5): (1) the *cytotoxic granule-associated RNA binding protein* (*TIA1*) gene that is involved in the negative regulation of cytokines (GO:0001818), negative regulation of translation (GO:0017148) and regulation of mRNA splicing (GO:0048024), and (2) the *interleukin 18 receptor* (*IL18R1*) gene that has a role in natural killer cell activation (GO:0030101), positive regulation of interferon-gamma production (GO:0032729) and cell signalling and inflammatory processes (KEGG: oas04060).

#### OAR5

On OAR5, we detected three QTL for *Prevotella* OTU1843, *Rikenellaceae_RC9_gut_group* OTU546 and *Ruminococcus* OTU1191. The QTL for OTU1843 includes two significant SNPs, of which one (rs402307868) showed a colocalized signal with the *Possible_genus_sk018* OTU1631. The allele substitution effects for the SNP with colocalized signals showed contrasting signs for the *Prevotella* and *Possible_genus_sk018* OTU. The QTL for *Rikenellaceae_RC9_gut_group* OTU546 includes two significant SNPs in high LD (LD score of 0.74). The QTL for *Ruminococcus* OTU1191 includes two significant SNPs separated by 0.04 Mbp, but the LD score was very low in that region (< 0.10). The SNP rs418870684 was associated with the novel gene *ENSOARG00000025325*.

#### OAR6

On OAR6, we detected six significant SNPs for five OTU but no QTL. In the region where a significant SNP was detected for alpha-S2-casein (81.2 Mbp), we identified two significant SNPs for *Acetitomaculum* OTU612, however, the LD score was low (< 0.20), and no shared SNPs were detected for these two traits.

#### OAR10

On OAR10, we detected a QTL with two significant SNPs at 16.2 Mbp for OTU1386 belonging to the order *Clostridia UCG-014*. The two significant SNPs were located in a region in high LD (LD score of 0.78), and the same gene, *E3 ubiquitin-protein ligase* (*W5PCZ6_SHEEP*), was detected. The *W5PCZ6_SHEEP* gene is involved in the ubiquitin-dependent protein catabolic process (GO:0006511), multicellular organism development (GO:0007275) and regulation of protein stability (GO:0031647).

#### OAR11

On OAR11, we detected a QTL with two significant SNPs in complete LD for *CAG-352* OTU304 belonging to the *Ruminococcaceae* family. However, this QTL for OTU304 was located far from the QTL detected for alpha-lactalbumin on OAR11 (32.6 to 34.2 Mbp). The candidate gene *helicase with zinc finger* (*HELZ*), that was located in this QTL, was not associated with any GO biological process or KEGG pathway.

#### OAR15

On OAR15, we detected the same significant SNP, rs409362751, for three *Prevotella* OTU (Table 5), and the allele substitution effects varied in the same way for the three *Prevotella* OTU. For this SNP, we identified the candidate gene *phosphodiesterase 3B* (*PDE3B*), which is involved in the negative regulation of cell adhesion (GO:0007162), negative regulation of lipid catabolic process (GO:0050995), and metabolic pathways (KEGG: oas01100).

#### OAR26

On OAR26, we detected the same significant SNP, rs412469804, for *Prevotella* OTU1336, *Prevotellaceae_UCG-001* OTU906 and OTU167 that belongs to the *F082* family. The allele substitution effects for the two *Prevotellaceae* OTU varied in the same direction, but in opposite directions for *F082* OTU167.

**Fig. 5:**
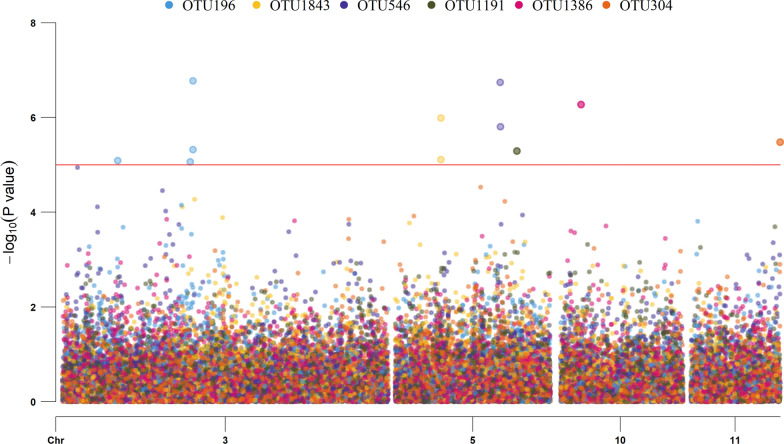
Manhattan plots for the genome-wide association studies of rumen bacterial abundance showing quantitative trait loci (QTL) on chromosomes 3, 5, 10 and 11. The horizontal red line shows the false discovery rate threshold of 0.10. Operational taxonomic units (OTU): *Prevotella* OTU196, *Prevotella* OTU1843, *Rikenellaceae_RC9_gut_group* OTU546, *Ruminococcus* OTU1191, *Clostridia UCG-014/*unknown family/unknown genus OTU1386, and *CAG-352* OTU304

As presented above, four SNPs on OAR3, 5, 15 and 26 simultaneously affected the rumen abundance of OTU belonging to the *Prevotellaceae*, *F082*, and *Lachnospiraceae* families (Fig. 5). However, no shared genomic regions were detected with the 18 dairy traits included in our study.

## Discussion

In this study, we hypothesised that, at least in part, the rumen microbiota abundance of Lacaune dairy ewes is under the control of the host genome and genetically linked with dairy traits through shared genomic regions coding for common metabolic pathways.

### Heritability of rumen bacteria and dairy traits

Our results obtained in Lacaune dairy ewes demonstrate that rumen bacterial abundance is partially controlled by the host genetics. The OTU heritabilities showed an asymmetric distribution with most values close to 0, but with 15% of the OTU (306 of 2059 OTU) having a low to moderate heritability (0.10 to 0.29), which is similar to the results reported for rumen bacteria in other ruminants [2, 4, 6, 42, 43]. However, there are some differences in the heritability range and in the percentage of OTU with a significant heritability. For example, Difford et al. [2] and Zhang et al. [6], using the same dataset of 750 dairy cows, obtained significant heritability estimates for 5 to 10% of genera, with heritabilities ranging from 0.17 to 0.25, and 2 to 6% of OTU with heritabilities ranging from 0.16 to 0.44. These results, which were based on 16S rRNA gene sequencing data, are similar to the range of heritabilities that we found for OTU, but the authors obtained a lower percentage of OTU with a significant heritability. It is also relevant to highlight the differences that were observed by working at different taxonomic levels, i.e., these authors reported lower heritabilities at the genus level. However, Martínez-Álvaro et al. [43], using untargeted “shotgun” sequencing, obtained significant heritabilities for 16% of genera and a range of higher heritabilities, with a maximum heritability of 0.60.

The group of 306 heritable OTU belonged to the most abundant phyla, such as *Bacteroidota* and *Firmicutes*, as reported by Li et al. [4], Wallace et al. [42] and Zhang et al. [6]. Furthermore, in terms of genus representation, *Prevotella*, which was overrepresented among the OTU with a significant heritability and showed an average estimate of 0.15 ± 0.04, is considered as a highly heritable genus in the literature [4, 42]. The main differences in terms of affiliation of the heritable rumen bacteria are with the results presented by Martínez-Álvaro et al. [43], with *Proteobacteria*, *Actinobacteria* and *Firmicutes* being the main represented phyla, but these differences could be due to the “shotgun” sequencing technique that reveals all the microbial genomes within a sample.

In general, the differences in gut bacteria observed between studies can be due to several factors: (1) the sequencing technology used, which does not give enough information to obtain affiliations at lower taxonomic levels (e.g., species), meaning that most studies report results at the genus level [2, 4, 6, 42] and rarely at the OTU level as we have in our study; (2) bioinformatic processing of DNA sequences and the subsequent analyses that have an impact on the total number of OTU to be analyzed and their taxonomic affiliations (e.g., Difford et al. [2] and Li et al. [4] amplified the V1–V3 region of 16S rRNA gene, meaning that some of their OTU were not obtained in our study because we amplified the V3–V4 region); (3) the number of individuals included in the analyses, which usually affects the accuracies of heritability estimates and explains the differences with studies that analyze fewer animals [43]; and (4) the animal species (bovine vs. sheep) investigated, which have differential bacterial abundances, as evidenced by Henderson et al. [44]. Notably, and in spite of the differences observed with other studies in ruminants, we report the highest percentage of bacterial OTU that are genetically controlled by the host.

However, a core microbiome cannot be declared based on these heritable OTU only, as was done by Wallace et al. [42], because we obtained only 44 of the 306 OTU present in most animals, and the same percentage of OTU with a significant heritability was observed in the core and total microbiotas. This result is expected because the core microbiome consists of microbes that are stable between animals in the same environment, which implies potential horizontal transmission of microbes between them. Therefore, our finding that only 14% of the host-genetically controlled OTU were part of the core microbiome means that most microbes come repeatedly from the environment and much less by vertical transmission from their parents.

To the best of our knowledge, our study is the first to estimate the genetic parameters of rumen bacteria from approximately 800 dairy ewes from the same farm under the same housing conditions.

For daily milk traits, we obtained moderate to high heritabilities, with values of 0.28 ± 0.06 for MY, 0.57 ± 0.06 for PC and 0.59 ± 0.06 for FC. Compared to two previous studies [7, 8] that include larger populations of Lacaune ewes in their first lactation, our estimates were similar to those of Rupp et al. [8] for MY (0.28) and PC (0.51) but higher for FC (0.41) on an annual basis, and to those of Boichard et al. [7] (PC 0.39 and FC 0.29) on a daily basis.

For the milk composition traits, milk FA presented moderate to high heritabilities, as reported by Boichard et al. [7] in Lacaune ewes. In addition, SFA had higher heritabilities than UFA, as reported by Boichard et al. [7] and by Buitenhuis et al. [3] who measured milk FA by accepted wet-lab methodologies in dairy cows. Milk proteins showed moderate (0.36 ± 0.08 for alpha-lactalbumin) to high (0.68 ± 0.07 for alpha-S2-casein) heritabilities, and were slightly higher than those reported in Lacaune ewes [7]. The heritability estimate for alpha-lactalbumin was low, as in Boichard et al. [7], which may be due to the low accuracy of this trait based on MIR spectrum prediction, with an R^2^ of 0.26 [20]. Thus, the results for alpha-lactalbumin should be interpreted with caution.

### Genetic correlations between rumen bacteria and dairy traits

Rumen bacteria are undeniably of crucial importance to their host, providing FA and microbial amino acids that contribute to milk fat and protein production [45]. However, only 96 heritable OTU from the most abundant families *Prevotellaceae*, *Lachnospiraceae* and *Ruminococcaceae* were moderately to highly genetically correlated (absolute values ranging from 0.33 to 0.99) with one or more milk FA and proteins. The overall quantification of these correlations is of interest to understand the relationship between rumen microbiota and phenotypes, in spite of the limitations of our study due to the low precision of 16S rRNA gene sequencing at the species level and the large number of estimated genetic correlations (more than 5500) based on a small dataset of 800 ewes, which, even if those with large standard errors are discarded, may lead to some spurious correlations.

The impact of the heritable OTU on the dairy traits was weak, as evidenced by the small number of significant genetic correlations obtained in our study (on average 301), i.e., only 5% of the total estimates. This figure was even smaller when we evaluated the number of OTU that were significantly associated with dairy traits in the core microbiome since only 13 of 96 OTU were genetically linked. However, interestingly, some of these OTU might have host-relevant functions since they are part of the core microbiome. Although the number of genetic correlations between rumen bacteria and milk traits was small, their values were moderate to high, which suggests that it could be useful to include microbiota composition in the genetic models, when the objective is to determine how the genetic effect impacts the phenotype not directly but with an indirect effect through microbiota abundance. In this regard, some authors have proposed methodologies to account for this indirect genetic effect mediated by the microbiota [46–48].

Our results show that most of the heritable OTU do not impact dairy traits. For this reason, the genetic associations that are observed mainly with milk FA and proteins can be difficult to use as selection criterion to improve fine milk composition traits, but this does not limit their inclusion in genetic models as an additional source of information to estimate the indirect genetic effect on those phenotypes of interest.

### GWAS of dairy traits and rumen bacteria

After having demonstrated that a group of heritable OTU was genetically linked to milk composition traits, we performed GWAS to identify host genomic regions that simultaneously affect rumen bacterial abundance and dairy traits. In the GWAS of dairy traits, we detected 22 significant SNPs that were distributed across nine chromosomes. Among these regions, two on OAR6 showed associations with alpha-lactalbumin and alpha-S2-casein. The SNP rs423428574 detected for alpha-S2-casein was located in the region of OAR6 where the major gene *CSN1S2* was reported in the literature on caseins [10, 11, 49]. In addition, in Lacaune sheep, Boichard et al. [7] detected a QTL on OAR6 for alpha-S2-casein, but without reporting a position. We have found no publications of QTL detected on OAR6 for alpha-lactalbumin in dairy ewes. On OAR11, we detected a QTL for alpha-lactalbumin with eight significant SNPs (Fig. 3), where a significant region for caseins and beta-lactoglobulin was also reported [7]. Although the major gene *LALBA*, which encodes alpha-lactalbumin in sheep, was identified on OAR3 [11], we found no significant SNPs for this whey protein in our study. Significant SNPs detected for alpha-lactalbumin on OAR2 and 20 were close to those that García-Gámez et al. [11] reported to be associated with protein and fat yields for dairy sheep. For the milk SFA C6:0 and C8:0, we detected the same SNP on OAR17, where one suggestive SNP was also identified for C4:0. García-Gámez et al. [11] reported a significant SNP (58.8 Mbp) on OAR17 that was associated with fat percentage, and Carta et al. [9] detected a QTL in the back-cross population of Lacaune sheep for C6:0 and C8:0 on OAR17 and one additional signal that we did not detect on OAR8. In addition, although we did not detect SNPs for milk FA on OAR11, Marina et al. [10] reported major genes for milk fat synthesis (*ACACA* and *FASN*) on this chromosome.

In the GWAS of rumen bacterial abundances, we detected signals that were distributed across the host genome, as reported by Li et al. [4], Abbas et al. [50] and Zhang et al. [6]. However, we detected three main regions on OAR3, 15 and 26 with signals for *Prevotellaceae* OTU that colocalized with the same SNP, i.e., colocalizations for *Prevotellaceae* and *Lachnospiraceae* OTU on OAR5, and colocalizations for *Prevotellaceae* and *F082* OTU on OAR26. This suggests that some regions of the host genome are associated with rumen bacterial abundance, but given the limitations raised by Pérez-Enciso et al. [51] about the difficulty in identifying causative SNPs for microbiota abundance, the results should be confirmed with a larger dataset. From the genomic regions in which QTL were detected for OTU abundances, we recovered several potential candidate genes, such as *TIA1*, *IL18R1*, *W5PCZ6_SHEEP* and *PDE3B*, which are involved in host immune system processes, regulation and catabolic processes, as reported by Abbas et al. [50]. However, it was not possible to identify shared metabolic pathways between OTU and dairy traits because we did not detect genomic regions where pleiotropic or closely-related QTL affected both traits simultaneously. One explanation may be that although the dataset is large for a microbial analysis, the number of records is too small for the detection of genome-associated regions and pleiotropic effects compared to other studies [52, 53].

## Conclusions

Our findings, based on a large and unique microbiome database, demonstrate that a small proportion of the total bacterial abundance in the rumen of Lacaune ewes is partially controlled by the host genome, for both the total and core microbiome, and that a few of the bacteria are associated with specific genomic regions on OAR3, 5, 15 and 26. Only very few of the genetic correlations between milk fine composition and rumen bacteria were significant, and it was not possible to identify genomic regions and metabolic pathways shared between rumen bacterial abundance and dairy traits. The use of 16S rRNA gene sequencing in this study did not allow for species-level affiliation, and additional work is needed to identify which microbial functions are involved and to associate the functions with milk traits. Then, it will be possible to consider the incorporation of microbiota abundance and microbial functions into genetic evaluation models to account for indirect genetic effects through the microbiota.

## Supplementary Information

**Additional file 1: Table S1.** Operational taxonomic unit (OTU) sequences with corresponding taxonomy affiliation. Consensus sequences of the OTU with corresponding affiliation from Kingdom to species.

**Additional file 2: Table S2.** Heritability (${h}^{2}$) and genetic correlations (rg) between lactation somatic cell score (LSCS), coefficient of variation in milk production (CV Milk), and operational taxonomic units (OTU). Heritability and genetic correlations for all 2059 OTU abundances with lactation somatic cell score and the coefficient of variation in milk production.

**Additional file 3: Table S3.** Genetic correlations between fat content (FC), milk fatty acids and operational taxonomic units (OTU). Genetic correlations between OTU abundances, fat content and milk fatty acids, such as butyric acid (C4:0), caproic acid (C6:0), caprylic acid (C8:0), capric acid (C10:0), lauric acid (C12:0), and palmitic acid (C16:0), oleic acid (*cis-9* C18:1), rumenic acid (*cis-9 trans-11* C18:2) and alpha-linolenic acid (C18:3*n-3*).

**Additional file 4: Table S4.** Genetic correlations between protein content (PC), milk proteins and operational taxonomic units (OTU). Genetic correlations between OTU abundances, protein content and milk proteins, such as alpha-S1-casein, alpha-S2-casein, beta-casein, kappa-casein, alpha-lactalbumin and beta-lactoglobulin.

**Additional file 5: Table S5.** Significant SNPs from GWAS of operational taxonomic units (OTU). Significant SNPs from GWAS of operational taxonomic units with candidate genes detected for each SNP.

**Additional file 6: Table S6.** Functional annotation for gene IDs recovered from detected SNPs for microbiome GWAS. Description of the functional annotations for genes recovered from Ensembl platform and metabolic processes and KEGG pathways from DAVID platform.

## Data Availability

The dataset supporting the conclusions of this article is available in the NCBI repository, https://www.ncbi.nlm.nih.gov/sra/PRJNA723543.
